# Hierarchical clustering of the pre-exam anxiety levels in physically inactive and active adolescent students from 56 countries: an observational study using PISA program data

**DOI:** 10.3389/fspor.2025.1509959

**Published:** 2025-07-07

**Authors:** Samuel Encarnação, José Eduardo Teixeira, Pedro Forte, Luciano Bernardes Leite, Andrew Sortwell, Luís Branquinho, Ricardo Ferraz, Pedro Afonso, Paula Vaz, António Miguel Monteiro

**Affiliations:** ^1^ Department of Sports Sciences and Physical Education, Instituto Politécnico de Bragança, Bragança, Portugal; ^2^ Department of Physical Education, Sport and Human Movement, Universidad Autónoma de Madrid (UAM), Ciudad Universitaria de Cantoblanco, Madrid, Spain; ^3^ Research Centre for Active Living and Wellbeing (Livewell), Instituto Politécnico de Bragança, Bragança, Portugal; ^4^ Department of Sports Sciences, Polytechnic of Guarda, Guarda, Portugal; ^5^ Department of Sports Sciences, Polytechnic of Cávado and Ave, Guimarães, Portugal; ^6^ SPRINT—Sport Physical Activity and Health Research & Innovation Center, Guarda, Portugal; ^7^ Research Center in Sports Sciences, Health Sciences and Human Development (CIDESD), Covilhã, Portugal; ^8^ CI-ISCE, Instituto Superior de Ciências Educativas do Douro (ISCE Douro), Penafiel, Portugal; ^9^ Department of Sports Sciences, Instituto Politécnico de Bragança, Bragança, Portugal; ^10^ Department of Physical Education, Universidade Federal de Viçosa, Viçosa, Brazil; ^11^ School of Health Sciences and Physiotherapy, University of Notre Dame Australia, Sydney, NSW, Australia; ^12^ Biosciences Higher School of Elvas, Polytechnic Institute of Portalegre, Portalegre, Portugal;; ^13^ Life Quality Research Centre (CIEQV), Portalegre, Portugal; ^14^ Department of Sports Sciences, University of Beira Interior, Covilhã, Portugal; ^15^ Department of Sports Sciences, University of Trás-os-Montes e Alto Douro, Vila Real, Portugal; ^16^ Centro de Investigação em Educação Básica (CIEB), Instituto Politécnico de Bragança, Bragança, Portugal

**Keywords:** anxiousness, unsupervised machine learning, teens, healthy lifestyle, social protection

## Abstract

**Introduction:**

The relationship between physical activity and anxiety among students has been extensively studied, with research highlighting the protective effects of physical activity on mental well-being.

**Methods:**

This article synthesizes existing literature on the topic and presents a novel analysis of pre-exam anxiety rates among physically inactive high school students from 56 countries. Using data from the Programme for International Student Assessment (PISA) 2018, a hierarchical clustering method was applied to identify four clusters based on stratified country groups by the students' pre-exam anxiety levels.

**Results:**

The results indicated five clusters for low physically active students (three with higher anxiety rates and two with lower levels of the condition) and four clusters for the low physically active individuals (two for higher anxiety rates and two for lower levels). Furthermore, the hierarchical model worked with good precision in the clustering task. In conclusion, considering the low physically active students, Brazil (82%) and the Dominican Republic (81%) recorded the highest pre-exam anxiety levels, while the Czech Republic (35%) had the lowest. Among the physically active students, Malaysia (82%), Brazil (81%), and Costa Rica (81%) recorded the highest anxiety levels, whereas again, the Czech Republic (35%) had the lowest.

**Discussion:**

These findings emphasize that although physical activity generally relates to reduced anxiety, this association varies across cultural and educational contexts.

## 1 Introduction

The relationship between anxiety and physical activity has been the subject of extensive research, with numerous studies highlighting the interplay between these two factors (1–6). To this end, the current evidence has consistently demonstrated the beneficial effects of physical activity on reducing symptoms of anxiety and improving mental well-being in various populations (7–10). A meta-analysis by Rebar et al. (11) emphasized the antidepressive and anxiolytic effects of physical activity, particularly in non-clinical adult populations.

The impact of physical activity on anxiety has also been studied in specific populations, such as students, demonstrating a negative relationship between physical activity and anxiety among secondary school students, indicating the potential benefits of a physically active lifestyle for the students' mental health (12). From a psychological perspective, Kayani et al. (13) suggested that the relationship between physical activity and academic anxiety is mediated by psychosocial factors, highlighting the complex interplay between these variables. Even with this complexity in mind, the prevalence of physical inactivity and anxiety among students still has significant implications for public health, mental well-being, and academic performance (13–15), and it has aroused interest in this field of research. As an example of this, Tabor et al. (16) investigated the negative relationship between smartphone usage and physical inactivity, emphasizing the importance of physical activity in managing anxiety (17). Moreover, Chao et al. (65) found that higher levels of physical activity were associated with lower risks of experiencing anxiety symptoms among Chinese college students, highlighting the global relevance of this relationship. Additionally, Wiet et al. (18) inferred from their study that physical activity is more effective than no treatment and as effective as traditional forms of treatment, including cognitive therapy and antidepressant medication, in addressing anxiety (19, 20).

In support and affirming of the evidence, the Association of Southeast Asian Nations (ASEAN) region has highlighted that the prevalence of anxiety among students has been linked to lifestyle factors, including physical inactivity and sedentary behavior (21). This link to lifestyle factors underscores the need to address not only the psychological aspects but also the lifestyle correlates of anxiety among students. The prevalence of physical inactivity and anxiety among students has been studied in various contexts, including during the COVID-19 pandemic, where the incidence of anxiety has been found to exceed that of the general population significantly (22, 23). These findings emphasize the need to understand the interplay between environmental, societal, and individual factors contributing to physical inactivity and anxiety among students. The research gap in the existing literature is evident in the limited attention given to physically inactive students from countries across all continents regarding their anxiety rates. While numerous studies have explored the relationship between physical activity and anxiety among students in specific regions or cultural contexts (24), there is a lack of comprehensive research focusing specifically on physically inactive students on a global scale. The existing body of research tends to focus on active populations or localized settings, leaving a crucial need for broader, more inclusive studies that address the unique experiences and challenges faced by inactive students worldwide (1, 2, 14, 16). This gap underscores the importance of understanding how inactivity influences mental health across diverse cultural, social, and geographical landscapes.

Identifying countries with higher rates of physical inactivity and anxiety among students is crucial for targeting vulnerable populations and developing effective interventions to promote physical activity and prevent anxiety-related issues (25). By focusing on these high-risk groups, we can develop more tailored approaches to safeguard students' mental health and foster academic success. In this context, employing methods that yield precise, actionable insights is particularly valuable, as they enable the creation of time-efficient interventions that can mitigate anxiety and support well-being. This study, therefore, implements a hierarchical clustering method to categorize countries based on students' pre-exam anxiety levels, specifically distinguishing between two subgroups: physically active and inactive high school students.

## 2 Methodology

### 2.1 Participants

Data from pre-exam anxiety levels of 600,000 scholar students, with an average age of 15 years, from 56 countries were recorded. The dataset was obtained from the Program for International Student Assessment (PISA) 2018 study (26). This initiative evaluated the ability of 15-year-old students to use their reading, mathematics, and scientific knowledge to meet real-life challenges. Therefore, the PISA also considered psychometric factors in their data collection (26, 27). Additionally, we included in the analysis the variables related to pre-exam anxiety levels and habitual physical activity classification in these students. We included the dataset containing percentual anxiety rates among students in each of the 56 countries by physical activity status: low physical activity, those students who do not engage in any regular physical activity, and high physical activity, those who regularly engage in moderate to vigorous physical activities. The sponsors for the data collection also included only anxiety and physical activity levels in subjects who reported feeling well-prepared for their exams to ensure that academic preparation was not a weighted influencing factor (23, 26). The dichotomous grouping (low vs. high physical activity) was guided by PISA's operational definition and common classifications in public health literature.

### 2.2 Data preparation and preprocessing

To cluster the adolescent's pre-exam anxiety levels by their physical activity levels, we implemented hierarchical clustering (HC), which is an unsupervised machine learning method that can identify patterns of dominance between factors and, thus, determine some outcome (dependent variable) of interest, and an independent variable (explanatory variable) (28). This algorithm is recognized to be useful in identifying unseen or without pre-defined categories present in a dataset. For this purpose, the HC works by merging smaller clusters into larger ones and splitting the larger ones into smaller ones; this method is considered an agglomerative approach (28, 29). This way, the data were prepared considering that the pre-exam anxiety levels were encoded in a numeric and continuous variable as the dependent variable (target) and the countries (independent variable) as the categorical variable. Each observation representing anxiety by its respective country was treated as an individual point, thus allowing the model to define these points in each cluster as more representative (29). The whole process was repeated two times for the anxiety levels by low or high physical activity levels; thus, two data frames were created: 1° [countries (independent variable)—pre-exam anxiety levels for low physical activity levels (dependent variable)] and 2° [countries (independent variable)—pre-exam anxiety levels for high physical activity levels (dependent variable)].

### 2.3 Hierarchical clustering algorithm implementation

To cluster the pre-exam anxiety levels based on the countries, the HC algorithm calculates Euclidean distance, which is basically the distance in multiple pairwise comparisons between all countries simultaneously (29). For better comprehension, the Euclidean distance is expressed in the following equation:

$$d(A,B)=\sqrt{\overset{n}{\underset{i=1}{\sum }}\;{\left(x_{A_{i}}-X_{b_{i}}\right)}^{2}}$$

where:

A*_ix_*_and B*_ix_* are the values of the *i*-th feature for countries A and B, respectively.
*n* is the number of features [e.g., pre-exam anxiety level in the present study (it can be more than one feature if applicable)].
The result gives the straight-line distance between countries A and B in a multidimensional feature space.

After applying the HC, following the review of clustering guidelines suggested by Gao et al. (28) to define the best number of clusters, we followed three sequential steps; first, building a dendrogram with the Euclidean distance values that allowed the visualization of all clusters and tracing of a horizontal line intersecting with the *y*-axis to define the most relevant number of clusters or those that comported the smaller clusters and split larger clusters in the same time; second, using the silhouette score to validate the ideal number of clusters (30); and third, evaluating the intra- and inter-dispersion of the chosen number of clusters with three metrics, i.e., within-cluster sum of squares (WCSS), between-cluster sum of squares (BCSS), and BCSS/WCSS ratio (31). Each of the prior steps is described below.

### 2.4 Performance evaluation

#### 2.4.1 Silhouette score

The silhouette score is a metric regarding the similarity level of a point with its cluster when fitting the comparison with other clusters, giving a notion of the internal cohesion and separation between clusters (30). This metric has a threshold of −1,1. To evaluate the goodness of the silhouette score, we considered the recommendations of Milligan and Cooper (32) that suggest a threshold of at least 0.5 or higher indicates a good clustering task, with a higher coefficient as the best number of clusters. For better comprehension, the silhouette score *s*(*i*) is shown in the following equation:

$$s(i)=\;\frac{b(i)-a(i)}{\max\;(a(i),b(i))}$$

where:

*a*(*i*) is the average distance between point *i* and all other points in the same cluster.
*b*(*i*) is the average distance between point *i* and all the points in the nearest cluster (the second closest cluster to which the point does not belong).

#### 2.4.2 Within-cluster sum of squares

The WCSS is the metric of intra-cluster variability, which calculates the square sum of the distances between points within their proper cluster and in relation to their centroid. The smaller the WCSS, the more approximated the points to the centroid of a cluster (31). The WCSS is expressed in the following equation:

$$\mathrm{WCSS}=\;\overset{K}{\underset{K=1}{\sum }}\;\underset{i\in C_{K}}{\sum }∥X_{i}-\mu_{k}{∥}^{2}$$

where:

*K* is the total number of clusters.
*C_k_* is the set of indices of the data points that belong to cluster *k*.
*x_i_* is the feature vector of data point *i*.
*μ_k_* is the feature vector of the centroid of cluster *k*.
∥*x_i_*−*μ_k_*∥² is the squared Euclidean distance between data point *i* and the centroid of cluster *k*.
∑*_i_*_∈*Ck*_ is the summation over all data points *i* that belong to cluster *k*.
∑*k* = 1 *K* is the summation over all clusters *k* from 1 to *K*.

In addition, to make the within-cluster approximations more interpretable, we divided the WCSS by the number of points (in our case, 56 countries), and thus we obtained the average square distance (31), as follows:

$$\mathrm{Average}\;\mathrm{square}\;\mathrm{distance}=\frac{\mathrm{WCSS}}{n\;\mathrm{points}}$$

After this, we calculated the square root of the average square distance, and we reobtained the percentual WCSS (31), which became fully interpretable in this context, better visualized in the following equation:

$$\mathrm{Percentual}\;\mathrm{WCSS}=\sqrt{\mathrm{Average}\;\mathrm{square}\;\mathrm{distance}}$$

#### 2.4.3 Between-cluster sum of squares

BCSS is a metric that measures the sum of the distances between the center of each cluster and the global centroid, weighted by the number of points in each cluster. Thus, BCSS measures between-cluster variation, or it gives the notion about the distance of each cluster centroid from another. The higher the BCSS score, the better separated the clusters are. In practice, the BCSS isolated is very valuable to understanding the level of separation of the clusters, but it also cannot isolate or evaluate the overall clustering quality, which is one more important step of the clustering (31). The BCSS equation is defined below:

$$\mathrm{BCSS}=\;\overset{k}{\underset{k=1}{\sum }}\;n_{k}∥\mu_{k}-\mu {∥}^{2}$$

As in WCSS, we followed some steps to make the index more interpretable. For this purpose, we normalized the BCSS by the initial number of selected clusters, having the average BCSS per cluster (31), better visualized in the equation:

$$\mathrm{Average}\;\mathrm{BCSS}\;\mathrm{per}\;\mathrm{cluster}=\frac{\mathrm{BCSS}}{n\;\mathrm{of}\;\mathrm{clusters}}$$

After this, we calculated the proportion of variability explained by the clustering by dividing the BCSS by the total sum of squares (TSS, the product of BCSS + WCSS). The higher the explained variability (0%–100%), the greater the HC capturing the variability within the data and inserting the points into the correct cluster (31). This is better explained by the following equation:

$$\mathrm{Explained}\;\mathrm{variation}=\frac{\mathrm{BCSS}}{\mathrm{TSS}}$$

The BCSS/WCSS ratio is applied to measure the relative separation between clusters in relation to the internal clustering comparison. This ratio completes the deficiencies of only using BCSS or WCSS isolated helping to holistically evaluate the quality of the clusters, because it can compare the variance between and within the clusters. This metric is more comprehensive and indicates whether the clusters are well-separated relative to the compactness of internal clusters. If a positive ratio is found, it can be considered that the HC is working well in separating the clusters and gives more confidence for the outputs (31). The BCSS/WCSS ratio can be understood by the following equation:

$$\mathrm{BCSS}/\mathrm{WCSS}\;\;\mathrm{ratio}=\frac{\mathrm{BCSS}}{\mathrm{WCSS}}$$

### 2.5 Statistical analysis

Since we had no access to the raw dataset, we assessed the prior calculation chi-square of two simple proportions (*χ*^2^) and its respective outputs (degrees of freedom, standard error of the mean, and *p*-values for a 95% confidence interval), already present in the Organisation for Economic Co-operation and Development web page (29). For this purpose, after the final fitted HC outputs, we reported the *χ*^2^ statistics (26). In addition, following the guidelines of The British Medical Journal (27), we used the available mean difference of the average percentual values for each country divided by the standard error of the difference: $\mathrm{Treshould}\;\mathrm{for}\;\mathrm{significnce}=\frac{\mathrm{Difference}\;\mathrm{between}\;\mathrm{means}}{\mathrm{Standard}\;\mathrm{error}\;\mathrm{of}\;\mathrm{the}\;\mathrm{difference}}$, thus obtaining the threshold corresponding to a difference above three standard deviations. In practical terms, a 99% confidence interval (*p* < 0.001) to reject the null hypothesis in which the two country means are equal (27, 28). The 20% *y*-axis threshold for cluster identification in the dendrogram was chosen based on heuristic precedent and previous recommendations for interpretability in similar large-scale clustering tasks (28). To test the robustness of this threshold, we performed a sensitivity analysis using alternate cutoffs (15% and 25%), which produced similar cluster compositions, validating the 20% choice.

## 3 Results

The HC algorithm identified two major clusters with subdivisions: first, five clusters [two clusters with high (H-1 and H-2) and three clusters with low anxiety rates (L-1, L-2, and L-3) for the low physically active students], and second, four clusters [two clusters with high (H-1 and H-2) and three clusters with low anxiety rates (L-1, L-2, and L-3) for the low physically active students]. The results of the analysis acknowledging low and highly physically active students and the algorithm performance after being tested by the evaluation metrics are described step-by-step below.

### 3.1 Students with low physical activity

**Figure 1:**
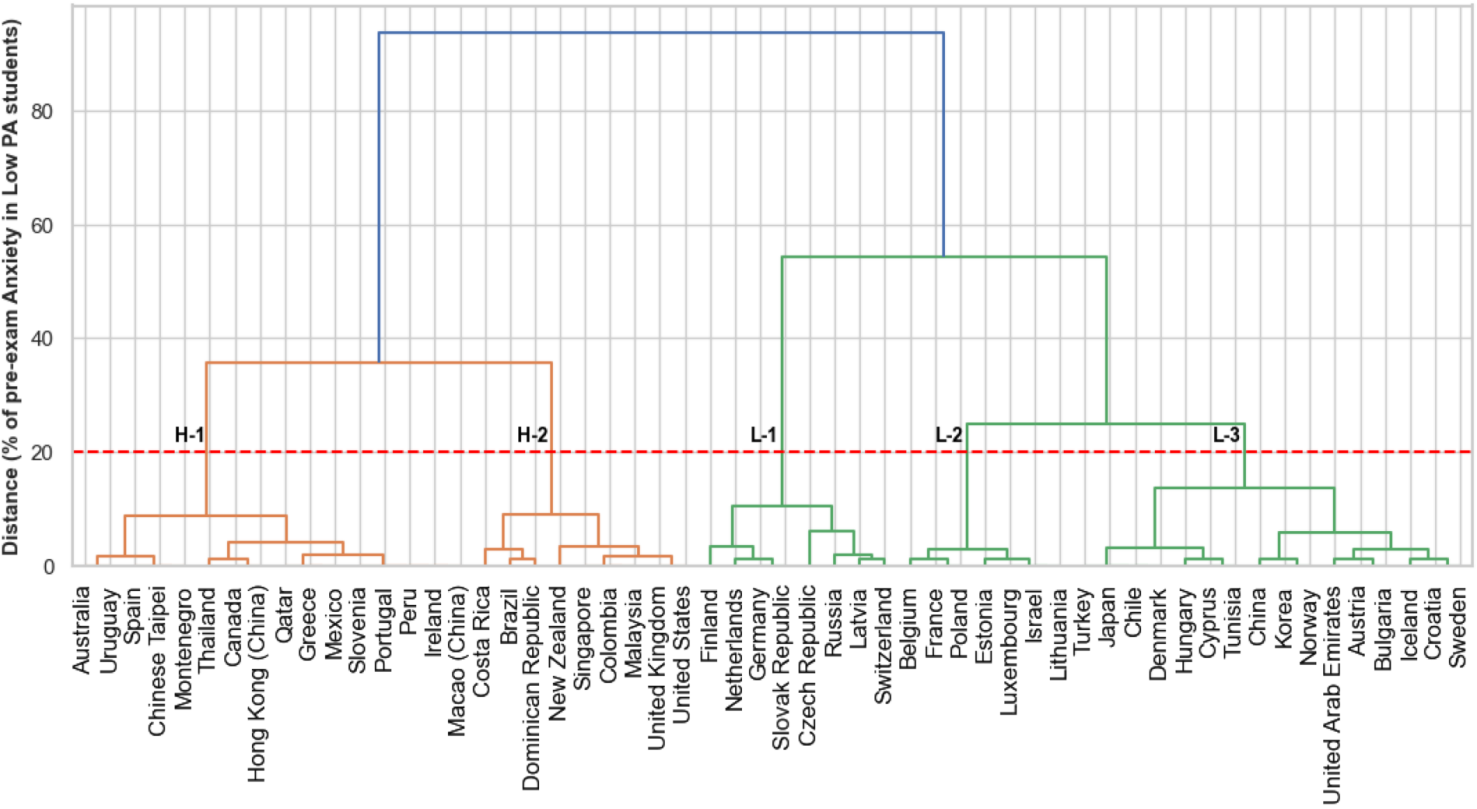
Dendrogram of the clustering for the pre-exam anxiety levels for high physical activity students across the countries. PA, physical activity. H-1 and H-2, clusters with the highest anxiety levels. L-1, L-2, and L-3, clusters with the lowest anxiety levels.

Figure 1 shows the results of the HC of anxiety levels among the 56 investigated countries considering the students classified with low anxiety levels. Following the step-by-step determination within the methods, the better number of clusters was considered when the horizontal lines intersected at 20% distance within the *y*-axis, followed by aggregation of five principal clusters, as observed in the red dashed line with the intersection with the five clusters' vertical lines. The two clusters in the orange line have the highest pre-exam average anxiety levels (H-1 = 67%, H-2 = 78%), and the three clusters in the green lines have lower average anxiety levels (L-1 = 41%, L-2 = 52%, L-3 = 60%). The height of the horizontal line of the clusters indicates the distance between the others; otherwise, there is a closed level of similarity between small clusters within the principal cluster. The best silhouette score (0.60) was in a combination of five clusters, considering a good number of clusters, WCSS = 309.27, with an average square distance of 5.52 for WCSS and a percentual average square distance of 2.35% indicating good compactness within the clusters, considering the range of 33%–82% of anxiety among the students. Furthermore, the BCSS analysis presents an absolute value of 1,367.33, with 96% of the variability explained by the clustering, showing very high precision in separating the clusters. Finally, the BCSS/WCSS ratio was positive (22.10), indicating good separation capacity between clusters compared with the variability within clusters.

Table 1 shows the pre-exam anxiety percentages for each country after HC results for the low physically active students. The clusters H-1 (67%) and H-2 (78%) presented higher pre-exam anxiety levels in the low physical activity students. Therefore, Brazil [H-2 (82%)] and the Dominican Republic [H-2 (81%)] had the most anxious pre-exam students. L-1 (41%), L-2 (52%), and L-3 (60%) represented the cluster with the smaller mean percentage of pre-exam anxiety, and the Czech Republic [L-1 (35%)] had less anxious students in the pre-exam moment. For students with low physical activity, L-1 (67%) and L-2 (78%) clusters were the most affected. It was perceptive that there was no predominance of a unique continent within the selected clusters.

**Table 1:** Clusters and respective percentage of pre-exam anxiety in low physically active students.

H-1 (67%)	H-2 (78%)	L-1 (41%)	L-2 (52%)	L-3 (60%)
Australia (70)	Brazil (82)	Finland (46)	Estonia (53)	Chile (63)
Uruguay (70)	Dominican Republic (81)	Netherlands (44)	Israel (52)	Denmark (63)
Chinese Taipei (69)	Colombia (79)	Germany (43)	Lithuania (52)	Japan (63)
Montenegro (69)	Costa Rica (79)	Slovak Republic (43)	Luxembourg (52)	Turkey (63)
Spain (69)	Malaysia (77)	Russia (41)	France (51)	Hungary (62)
Ireland (67)	Singapore (77)	Latvia (40)	Poland (51)	Cyprus (61)
Macao (China) (67)	United Kingdom (76)	Switzerland (39)	Belgium (50)	Tunisia (61)
Peru (67)	United States (76)	Czech Republic (35)		China (60)
Portugal (67)	New Zealand (74)			Korea (59)
Slovenia (67)				Norway (59)
Greece (66)				United Arab Emirates (58)
Qatar (66)				Bulgaria (57)
Hong Kong (China) (65)				Austria (57)
Canada (65)				Sweden (56)
Mexico (66)				Croatia (56)
Thailand (64)				Iceland (55)

Data are presented in mean percentual values for each country. H-1 and H-2, clusters with the highest anxiety levels. L-1 and L-2, clusters with the lowest anxiety levels.

### 3.2 High physical activity level students

**Figure 2:**
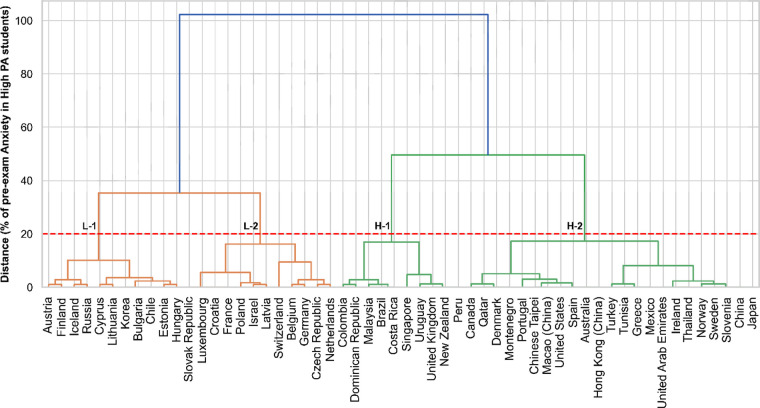
Dendrogram of the clustering for the pre-exam anxiety levels for high physical activity students across the countries. PA, physical activity. L-1 and L-2, clusters with the lowest anxiety levels. H-1 and H-2, clusters with the highest anxiety levels.

Figure 2 shows the results of the pre-exam anxiety levels among the 56 countries adjusted for the students with satisfactory physical activity levels. The better number of clusters was four, also reaching this performance when the horizontal line intersected 20% of the distance in the *y*-axis of the dendrogram, as observed through the dashed line intersecting with four vertical lines (four principal clusters). The two clusters with orange lines have the lowest pre-exam anxiety levels (L-1 = 53%, L-2 = 43%), and the clusters with green lines have higher pre-exam anxiety levels (L-1 = 77%, L-2 = 64%). The dendrogram for highly physically active students showed good similarity between the small clusters originating from the principal clusters. The silhouette method also had a good score (0.56), indicating that the clusters were also well separated for this analysis, followed by WCSS = 632.80, with an average square distance of 5.52 for WCSS, and a percentual average square distance of 11.3%, indicating satisfactory compactness within clusters considering the anxiety rate range of 33%–82% among the students. The BCCS results (7,079.18) also explained the variability between clusters when separating clusters (92%). The BCSS/WCSS ratio was also positive (11.18), showing that the clusters were also well-separated from the others when considering their internal variability.

Table 2 shows the pre-exam anxiety percentages by country after the HC application, considering the students' satisfactory physical activity levels. The L-1 (53%) and L-2 (43%) clusters exposed the lowest pre-exam anxiety levels. Switzerland had the lowest anxiety levels (33%) within the L-2 cluster. In contrast, the H-1 (77%) and H-2 (64%) clusters had the highest anxiety rates. Malaysia (82%), followed by Brazil (81%) and Costa Rica (81%), had the highest rates of anxiety within the H-1 cluster, and the Czech Republic (35%) had lower rates of anxiety among the physically active students. Considering physically active students, the H-1 (77%) and H-2 (64%) clusters identified the students who were more impacted by elevated rates of anxiety before their scholar examination. Like the analysis enrolling the physically inactive students, the clusters regarding the individuals in low physical activity levels also did not present a predominance of continents within the final selection of clusters.

**Table 2:** Clusters and respective percentage of pre-exam anxiety in physically active students.

L-1 (53%)	L-2 (43%)	H-1 (77%)	H-2 (64%)
Cyprus (57)	Croatia (47)	Malaysia (82)	Portugal (69)
Lithuania (56)	France (47)	Brazil (81)	Australia (67)
Bulgaria (55)	Luxembourg (47)	Costa Rica (81)	Hong Kong (China) (67)
Chile (55)	Slovak Republic (47)	Dominican Republic (80)	Spain (67)
Korea (55)	Poland (45)	Colombia (79)	United States (67)
Hungary (54)	Israel (44)	Singapore (76)	Chinese Taipei (66)
Estonia (53)	Latvia 43	Uruguay (73)	Macao (China) (66)
Russia (52)	Belgium 42	Peru (72)	Denmark (65)
Iceland (51)	Germany 41	New Zealand (72)	Montenegro (65)
Austria (50)	Czech Republic 40	United Kingdom (72)	Qatar (65)
Finland (49)	Netherlands 39		Canada (64)
	Switzerland 33		Ireland (63)
			Thailand (63)
			United Arab Emirates (63)
			China (62)
			Japan (62)
			Slovenia (62)
			Sweden (62)
			Norway (61)
			Greece (59)
			Mexico (59)
			Tunisia (59)
			Turkey (58)

Data are presented in mean percentual values for each country. L-1 and L-2, clusters with the lowest anxiety levels. H-1 and H-2, clusters with the highest anxiety levels.

Table 3 shows only the countries with significant mean percentual differences when comparing students with low- and high physical activity levels. The *χ*^2^ statistics calculated *a priori* with the raw data in the PISA study showed no differences between physical activity groups when considering the total number of observations (full sample) included in the study. However, when individually analyzing the differences in the percentual of anxiety by groups and considering the cutoff of *Z* ≥ 1.96, there were some countries with beneficial statistically significant reductions in the pre-exam anxiety levels, *p* < 0.05 [Austria (−7), Belgium (−8), Chile (−8), France (−5), Germany (−2), Greece (−7), Hungary (−7), Israel (−9), Korea (−5), Mexico (−7), Turkey (−5), United States (−9%), Croatia (−9%)]. There was only an exception for Russia (+11%) and the United Arab Emirates (+4%), which increased their levels of anxiety as the participants were highly physically active. Furthermore, the average of all countries was a significant average percentual change (−3%, *p* < 0.01), showing that the physical activity status was important to reduce pre-exam anxiety if the students were prepared for the exam. Finally, it was notable that those countries that benefited from a physically active lifestyle were those which were framed in clusters with the lowest rates [L = 12 (67%) vs. H = 3 (23%)] when the students were in low physical activity levels, or apparently, a physical activity lifestyle was more effective in reducing the levels of anxiety in those countries with prior reduce rates of the outcome than in those countries with the highest rates.

**Table 3:** Individual differences between the countries compared by physical activity status.

Country	Do not engage in any physical activity		Engage in physical activity (moderate or vigorous)		*χ*^2^ statistics				Threshold (95%—IC)
	% (cluster)	SE	% (cluster)	SE	*χ* ^2^	*p*	% Diff.	SE (diff.)	*Z*
Austria	57.2 (L-3)	2.42	50.2 (L-1)	0.86	0.18	0.67	−7**	2.4	−2.89
Belgium	49.9 (L-2)	1.94	41.8 (L-2)	0.62	0.33	0.57	−8***	2	−43
Chile	62.9 (L-3)	2.34	55.2 (L-1)	0.77	0.20	0.65	−8***	2.5	−39
France	51.3 (L-2)	2.21	46.5 (L-2)	0.80	08	0.78	−5*	2.4	−1.98
Germany	43.1 (L-1)	4.17	41.1 (L-2)	0.75	00	0.95	−2	4.4	−0.45
Greece	65.5 (H-1)	2.48	58.5 (H-2)	0.71	0.14	0.70	−7**	2.5	−2.75
Hungary	61.5 (L-3)	3.10	54.2 (L-1)	0.83	0.16	0.68	−7**	3	−2.41
Israel	52.2 (L-2)	2.53	43.5 (L-2)	0.73	0.39	0.53	−9***	2.6	−3.39
Korea	59.4 (L-3)	1.90	54.7 (L-1)	0.79	05	0.81	−5**	1.9	−2.43
Mexico	66.1 (H-1)	2.80	59.4 (H-2)	0.82	0.13	0.72	−7*	2.9	−2.32
Turkey	63.2 (L-3)	2.32	58.1 (H-2)	0.87	06	0.80	−5*	2.5	−25
United States	75.7 (H-2)	2.38	67.1 (H-2)	0.69	0.19	0.65	−9***	2.4	−3.64
Croatia	55.9 (L-3)	2.77	46.5 (L-2)	0.86	0.40	0.52	−9***	2.7	−3.46
Russia	40.6 (L-1)	4.40	51.5 (L-1)	0.80	0.66	0.41	11**	4.2	2.57
United Arab Emirates	58.2 (L-3)	1.21	62.7 (H-2)	0.70	043	0.83	4***	1.3	3.57
OECD average	57.7	0.51	54.8	0.13	01	0.91	−3***	0.5	−5.48

SE, standard error for individual groups; *χ*^2^, chi-square statistics; *p*, 95% CI *p*-value; %Diff., percentual difference; SE (diff.), standard error of the difference; Z, Z-statistics for a 95% CI.

* *p* < 0.05.

** *p* < 0.01.

*** *p* < 0.0001.

## 4 Discussion

The present study sought to identify patterns of pre-exam anxiety among high school students, distinguishing between those who engage in moderate or vigorous physical activity and those who are physically inactive. The results showed that countries with a higher incidence of physically inactive students generally exhibited higher levels of pre-exam anxiety compared with their physically active counterparts. This finding reinforces the extensive literature associating regular physical activity with psychological benefits, including the reduction of anxiety and stress symptoms.

Initially, in recent years, the practice of physical activity has been widely studied as a protective factor for mental health, particularly concerning anxiety (33, 34). Studies indicate that regular physical exercise can act as a modulator of the central nervous system, reducing cortisol levels, a hormone associated with stress (35–37), and increasing the release of neurotransmitters such as endorphins and serotonin, which promote feelings of well-being (38, 39). Initially, in recent years, the practice of physical activity has been widely studied as a protective factor for mental health, particularly anxiety (33, 34). Studies suggest that regular physical exercise can act as a modulator of the central nervous system, reducing cortisol levels, a hormone associated with stress (12, 36), and increasing the release of neurotransmitters such as endorphins and serotonin, which promote feelings of well-being (38, 40). These mechanisms partially explain why physically active students exhibit lower levels of anxiety, as evidenced by the results of the present study. However, it is important to note that, despite a general trend of lower anxiety levels among physically active individuals, this relationship was not universally observed across all samples. This suggests that, while physical activity is beneficial, other factors, such as the intensity of academic stress and expectations surrounding academic performance, may influence anxiety levels (41). Therefore, in such cases, exercise alone may not be sufficient to fully mitigate the impact of academic pressure on students' mental health. Emerging neuroimaging studies provide further insight into the relationship between physical activity and anxiety. Exercise has been shown to influence amygdala reactivity and prefrontal cortex function, enhancing emotional regulation and resilience.

Furthermore, it is plausible that the beneficial impact of physical activity on reducing anxiety is influenced by variables such as the type, frequency, and intensity of the exercise performed (42, 43). Generally, engaging in physical exercise, regardless of its intensity—moderate or high—is widely recognized as a contributing factor to decreased anxiety (44, 45). However, in highly competitive contexts, physical activity can, paradoxically, exacerbate anxiety levels (18, 46–48). In this sense, exercises conducted in non-competitive environments tend to be more effective in mitigating anxiety levels, while activities in competitive situations can intensify stress, especially in individuals who are already under high pressure (48, 49). The consistently low anxiety levels reported by Czech students, regardless of physical activity status, suggest that cultural and educational factors may play a protective role. The Czech education system is characterized by reduced high-stakes testing pressure compared with other countries, which may lower exam-related stress (50). Additionally, cultural attitudes toward well-being and academic resilience may buffer anxiety (51). In countries such as Brazil or Malaysia, the intensity of entrance examinations and competitive academic environments may exacerbate anxiety (23). In contrast, Scandinavian or Central European education systems often emphasize holistic development over performance metrics, potentially explaining the lower anxiety levels reported (52). In countries such as Costa Rica or the Dominican Republic, scalable community-based physical activity programs could be integrated into school curricula, emphasizing inclusive participation over performance (53, 54). Mental health literacy campaigns adapted to cultural norms could also help destigmatize anxiety and promote help-seeking behaviors (55). Thus, the variability observed in anxiety levels among physically active students may be associated not only with the types of exercise but also with the context in which they are performed, as well as the level of stress to which students are exposed.

Physically active students in countries such as Russia and the United Arab Emirates (UAE) often experience high anxiety due to academic and cultural pressures, despite the mental health benefits of exercise. Studies show elevated anxiety among UAE students, especially females during the COVID-19 pandemic (56), and among Russian medical students facing intense academic demands (57), where in these contexts, physical activity might be associated with performance pressure (e.g., competitive sports). While physical activity generally supports mental health, it may not offset anxiety when institutional stress and mental health stigma are present (39, 58–60). Regarding physically inactive students, the higher levels of anxiety observed in the study reinforce the literature highlighting the mental health risks of a sedentary lifestyle, especially among adolescents. Various studies demonstrate that sedentary behavior is associated with compromised mental health (61–64). Additionally, the lack of physical activity can significantly contribute to the worsening of anxiety, as sedentary individuals often have greater difficulty coping with stress and pressure situations (64). The absence of exercise can negatively affect emotional well-being, reducing the ability to face daily challenges, such as those related to the academic environment, which ultimately intensifies anxiety, especially during peak demand periods such as exams (61, 62).

## 5 Practical applications and limitations

Despite its promising findings, this study has some limitations that should be considered. First, the generalizability of the results may be restricted by the data source used, as the sample is limited to students who participated in the PISA study, which may not adequately represent all high school students in a global context. Moreover, the dataset considered the average of the percentual values of anxiety for each country, which could lose an essential variability component within each country, that could improve the clustering breakdown. Additionally, the study's focus on 15-year-old students may not encompass the full diversity of age groups and educational environments among high school students. It is also important to note that self-reported data may have introduced response biases, as the participants themselves assessed the levels of physical activity and anxiety. These biases may inflate the correlation between the two variables. The sample was selected based on its scope and standardization; PISA 2018 includes a broad spectrum of countries with consistent measures across multiple domains. However, this selection may not fully capture nuances in less-represented regions or local socioeconomic disparities, potentially limiting the generalizability of findings to underrepresent cultural or educational systems.

Although the study revealed significant associations between physical activity and anxiety levels, it is crucial to recognize that reducing anxiety through exercise should be understood as part of a broader approach to promoting mental health. Interventions that focus exclusively on physical activity may not be sufficient to effectively address the complexity of factors influencing pre-exam anxiety, especially in contexts where academic stress is high and emotional support is limited. Therefore, it is essential that physical activity is integrated with other forms of psychosocial support, such as counseling and stress management, in order to enhance the beneficial effects on students' mental health. The outputs of this study can be used by policymakers from the most affected countries by the issues and consequences of anxiety to the scholarly students' mental health in building educational projects and meetings with parents and teachers, which helps the scholarly community to be more conscious and empowered to prevent long-term anxiety levels and ensure good academic development for the most vulnerable students. While general recommendations are important, we recognize the need for context-specific strategies. For example, in Brazil—one of the countries with the highest anxiety levels—policymakers could consider mandatory inclusion of physical education (PE) at all high school levels, integrated with mindfulness and stress management curricula. Collaboration between schools and mental health professionals may also help develop anxiety screening protocols and physical activity interventions tailored to students' needs. For future research, similar clustering methods can be implemented in case studies in different realities, thus providing the opportunity to make rich insights when comparing the findings across the diversity of socioeconomic and demographic factors among students. The employment of similar models in other age groups, such as university students, can also add to the particularities of anxiety in different academic periods of a person’s life. Finally, longitudinal approaches incorporating time series analysis plus clustering can be very explanative of long-term changes in anxiety levels among students. Future studies should also consider incorporating objective measures (e.g., accelerometers and validated clinical anxiety inventories) to validate these findings.

## 6 Conclusions

We conclude that the hierarchical clustering presented good performance in highlighting the most anxious countries in pre-exam, adjusting the clustering by physical activity groups. The countries with respective low physically active students were clustered within five clusters, in which Brazil (82%) and the Dominican Republic (81%) had the most anxious pre-exam students and the Czech Republic (35%) had less anxious students at the pre-exam moment. Regarding the physically active students, these countries were grouped within four clusters, where Malaysia (82%), Brazil (81%), and Costa Rica (81%) were the most anxious countries and the Czech Republic (35%) had the lower rates of anxiety among the physically active students.

## Data Availability

Publicly available datasets were analyzed in this study. This data can be found here: https://www.oecd.org/en/data/datasets/pisa-2018-database.html#data.
